# Cannabidiol Affects the Bezold-Jarisch Reflex *via* TRPV1 and 5-HT_3_ Receptors and Has Peripheral Sympathomimetic Effects in Spontaneously Hypertensive and Normotensive Rats

**DOI:** 10.3389/fphar.2019.00500

**Published:** 2019-05-22

**Authors:** Rafał Kossakowski, Eberhard Schlicker, Marek Toczek, Jolanta Weresa, Barbara Malinowska

**Affiliations:** ^1^ Department of Experimental Physiology and Pathophysiology, Medical University of Białystok, Bialystok, Poland; ^2^ Department of Pharmacology and Toxicology, University of Bonn, Bonn, Germany

**Keywords:** cannabidiol, arterial hypertension, Bezold-Jarisch reflex, TRPV1 receptors, 5-HT_3_ receptors, sympathomimetic

## Abstract

Cannabidiol (CBD) is a nonpsychotropic constituent of *Cannabis sativa* L. It is suggested to be useful in hypertension. Under *in vitro* conditions, it activates vanilloid TRPV1 and inhibits serotonin 5-HT_3_ receptors, i.e., receptors involved in the Bezold-Jarisch reflex stimulation. The aim of our study was to compare the cardiovascular effects of CBD in spontaneously hypertensive (SHR) and normotensive Wistar Kyoto (WKY) rats. Experiments were performed on conscious, urethane-anesthetized, and pithed rats. In pithed SHR and WKY, CBD increased heart rate (HR) and systolic blood pressure (SBP) and decreased diastolic BP (DBP) in a manner insensitive to adrenalectomy. Propranolol strongly impaired the CBD-induced increases in HR and SBP without affecting the decreases in DBP. Desipramine also reduced the CBD-induced effects on HR and SBP and further increased its effects on DBP. In anesthetized rats, bolus *i.v.* injection of single doses of CBD induced short-lasting decreases in HR, SBP, and DBP, stronger in SHR than in WKY and prevented by bilateral vagotomy. The CBD-induced fall in HR but not in BP was diminished by the TRPV1 receptor antagonist capsazepine and almost completely abolished if CBD was re-injected after previous administration. CBD reduced the Bezold-Jarisch reflex elicited by the 5-HT_3_ receptor agonist phenylbiguanide but not that evoked by the TRPV1 agonist capsaicin. In conscious rats, CBD did not affect cardiovascular parameters. In isolated left atria, CBD decreased contractile force. Conclusions: Cannabidiol (1) induces the Bezold-Jarisch reflex likely *via* TRPV1 receptors (which undergo tachyphylaxis) more markedly in SHR than in WKY; (2) inhibits the Bezold-Jarisch reflex induced by activation of 5-HT_3_ but not TRPV1 receptors; (3) has peripheral sympathomimetic, (4) vasodilatory, and (5) negative inotropic effects. The above properties of CBD should be taken under consideration when CBD is used for therapeutic purposes.

## Introduction

Cannabidiol (CBD) is the main nonpsychoactive compound of marijuana. Its unique pharmacological properties make this compound highly attractive for therapeutic use. Its effectiveness against some types of epilepsy (Chen et al., 2019) and, in combination with Δ^9^-tetrahydrocannabinol (nabiximols), against symptoms associated with multiple sclerosis (Patti, 2019) is established. Moreover, preclinical and clinical studies indicate that CBD has a potential therapeutic activity also as an anxiolytic, antipsychotic, antidepressant, neuroprotective, or cerebrovascular blood-brain barrier protective agent (Booz, 2011; Pisanti et al., 2017; Ruggiero et al., 2017; Sultan et al., 2017; Peres et al., 2018; Brook et al., 2019).

CBD has also been suggested as a potential antihypertensive drug (Booz, 2011; Stanley et al., 2013; Sultan et al., 2017) mainly because of its direct vasodilator properties shown in isolated human (Stanley et al., 2015) and rat (Offertáler et al., 2003) mesenteric arteries and in rat aorta (O’Sullivan et al., 2009). CBD also improved the vasorelaxant response to acetylcholine in isolated mesenteric arteries from Zucker diabetic fatty rats (Wheal et al., 2017) and spontaneously hypertensive rats (SHR; Stanley et al., 2013).

Controversial effects on cardiovascular parameters were obtained with CBD in the rat *in vivo*. CBD did not modify resting blood pressure (BP) and/or heart rate (HR) in normotensive rats when administered (1) intraperitoneally (*i.p.*) to conscious rats (Resstel et al., 2009), (2) intravenously (*i.v.*) to urethane-anesthetized (Graham and Li, 1973) and pentobarbitone-anesthetized rats (McQueen et al., 2004), (3) into the bed nucleus of the stria terminalis (BNST; structure with a direct influence on autonomic functions) of tribromoethanol-anesthetized (Alves et al., 2010) and conscious (Gomes et al., 2013) rats, and (4) into the cisterna magna of pentobarbitone-anaesthetized rats (Granjeiro et al., 2011). By contrast, CBD given *i.p*. (Resstel et al., 2009) or into the cisterna magna (Granjeiro et al., 2011) reduced BP and HR in stressful conditions but its microinjection into the BNST increased HR to acute restraint stress (Gomes et al., 2013).

In humans, like in rats, cardiovascular effects of CBD are more evident in stressful conditions (Sultan et al., 2017). Thus, in healthy volunteers, a single dose of CBD 600 mg, which reduced resting BP and increased HR (Jadoon et al., 2017) or did not modify cardiovascular parameters (Martin-Santos et al., 2012) under standard conditions, blunted the increases in BP and HR elicited by cold stress (Jadoon et al., 2017). Moreover, CBD 400 mg decreased subjective anxiety in patients with social anxiety disorders, which was accompanied with changes in regional cerebral blood flow (Martin-Santos et al., 2012). Hypertension is connected with an increase in sympathetic tone (Saxena et al., 2018). However, any direct evidence for an antihypertensive activity of CBD is so far lacking.

CBD has a very low affinity to CB_1_ and CB_2_ receptors; it acts by multiple mechanisms (Pertwee et al., 2010). Among others, CBD has been shown to be a full, but not potent, agonist of human TRPV1 receptors in human embryonic kidney (HEK) 293 cells (Bisogno et al., 2001; De Petrocellis et al., 2011; Iannotti et al., 2014). These receptors are involved in the anxiolytic effect of CBD in rats (Campos and Guimarães, 2009), in its antihyperalgesic effect in a rat model of acute inflammation (Costa et al., 2004), and in its anti-inflammatory effect in the mouse experimental autoimmune hepatitis (Hegde et al., 2011). In addition, CBD inhibited human and mouse 5-HT_3A_ receptors in HEK293 cells (Xiong et al., 2011) and in *Xenopus laevis* oocytes (Yang et al., 2010). Both TRPV1 and 5-HT_3_ receptors are located on cardiac sensory vagal nerves and induce the Bezold-Jarisch reflex, i.e., a rapid reflex bradycardia and hypotension. We have previously shown that the endocannabinoid anandamide and its stable analogue methanandamide stimulated (Malinowska et al., 2001) and the synthetic cannabinoids WIN55212–2 and CP55940 inhibited (Godlewski et al., 2003) the Bezold-Jarisch reflex *via* stimulation of TRPV1 and inhibition of 5-HT_3_ receptors, respectively.

Thus, the aim of the present study was to examine the influence of CBD on BP and HR and on the TRPV1 and 5-HT_3_-receptor mediated Bezold-Jarisch reflex in spontaneously hypertensive and normotensive rats. Moreover, we studied its effects on cardiovascular parameters in the pithed rat and on the rat heart *in vitro*.

## Materials and Methods

### Animals

All surgical procedures and experimental protocols were performed in accordance with the European Directive (2010/63/EU) and Polish legislation and in compliance with the ARRIVE guidelines and the so-called 3Rs (replacement, refinement, or reduction). The study was approved by the local Animal Ethics Committee in Olsztyn (nr 40/2016, 80/2017). Rats were housed in plastic cages (two rats per cage) with sawdust on the bottom in a temperature-controlled room at 22 ± 1°C under a 12:12 h light–dark cycle with free access to water and standard laboratory food.

All experiments were performed on 8-to-10-week-old male spontaneously hypertensive rats (SHR; 270–320 g) and age-matched normotensive Wistar-Kyoto rats (WKY; 290–390 g).

### Telemetric Measurement of Cardiovascular Parameters in Conscious Rats

Implantable telemetry transmitters (HD-S10; DSI, St. Paul, MN, USA) were used to measure systolic (SBP) and diastolic (DBP) blood pressure and heart rate (HR) in conscious unrestrained rats as described previously (e.g., Froger-Colléaux et al., 2011). Briefly, rats were anesthetized with pentobarbitone sodium *i.p.* (300 μmol/kg; i.e., ~70 mg/kg). Then, catheters were implanted into the femoral artery, and the body of the transmitter was placed into a subcutaneous pocket. Rats were allowed to recover for 1 week before the measurements.

### Measurement of Cardiovascular Parameters in Conscious Rats by the Tail-Cuff Method

Prior to anesthesia, SBP and HR were recorded in conscious rats by the noninvasive tail-cuff method using the Rat Tail Blood Pressure Monitor from Hugo Sachs Elektronik-Harvard Apparatus (March-Hugstetten, Germany).

### Measurement of Cardiovascular Parameters in Anesthetized Rats

Rats were anesthetized with urethane *i.p.* (14 mmol/kg; i.e., 1,250 mg/kg). This anesthesia was sufficient until the end of experiments, and we did not observe any withdrawal reflexes elicited by paw-pinch. Following anesthesia, the animals were tracheotomized. The carotid artery was carefully separated from the vagus nerve and cannulated to measure SBP and DBP *via* a pressure transducer (“ISOTEC”; Hugo Sachs Elektronik, March-Hugstetten, Germany). The heart rate was recorded using an electrocardiogram. The body temperature was kept constant at about 36–37°C using a heating pad (Bio-Sys-Tech, Białystok, Poland) and monitored with a rectal probe transducer (RDT 100; Bio-Sys-Tech, Białystok, Poland). The left femoral vein was cannulated for *i.v.* administration of drugs at a volume of 0.5 ml/kg. After 15–30 min of equilibration, during which the cardiovascular parameters were allowed to stabilize, the experiments were performed. Some experiments were performed on anesthetized and vagotomized rats.

### Measurement of Cardiovascular Parameters in Pithed Rats

Urethane-anesthetized rats were pithed after cannulation of the trachea by inserting a stainless-steel rod (1.5 mm diameter and 190 mm length) through the orbit and foramen magnum into the vertebral canal. Pithed animals were artificially ventilated with air (1 ml/100 g, 60 strokes/min) using a respirator (7,025 Rodent respirator; Hugo Sachs Elektronik, March-Hugstetten, Germany). The body temperature was monitored with a rectal probe transducer (RDT 100; Bio-Sys-Tech, Białystok, Poland) and kept constant at about 36–37°C using a heating pad (Bio-Sys-Tech, Białystok, Poland). Both vagal nerves were cut. In some pithed rats, both adrenals were removed immediately after anesthesia and before pithing and vagotomy. For this purpose, the periadrenal tissue between the kidney and the adrenal was grasped using a small curved forceps, and the whole intact gland together with the periadrenal fat was removed.

### Experimental Protocol

#### Cardiovascular Effects of CBD in Conscious Rats

Seven days after transmitter implantation, CBD 10 mg/kg or its vehicle was infused *i.p.* over a time period of about 30 s and cardiovascular parameters were measured telemetrically for 1 h.

#### Cardiovascular Effects of CBD in Anesthetized Rats

Cannabidiol (3, 10 and 30 mg/kg i.e., ~ 9.5, 32 and 95 μmol/kg, respectively) or its vehicle was rapidly injected *i.v.* In order to construct the dose-response curve for CBD, each dose of CBD was examined in separate rats. Only in one series of experiments (dedicated to prove CBD-related tachyphylaxis), CBD 10 mg/kg was injected *i.v.* after previous *i.v.* administration of CBD 30 mg/kg with sufficient time for recovery to the pre-injection value. In additional experiments, CBD 10 mg/kg was examined (1) in vagotomized rats and (2) in the presence of the TRPV1 receptor antagonist capsazepine 1 μmol/kg (i.e., ~0.4 mg/kg; Malinowska et al., 2001) or its vehicle given 2 min earlier in order to determine its potential reflex response or the involvement of TRPV1 receptors, respectively.

#### Influence of CBD on the Bezold-Jarisch Reflex in Anesthetized Rats

The Bezold-Jarisch reflex was induced by rapid *i.v.* injection of the TRPV1 receptor agonist capsaicin (1–300 μg/kg; i.e., ~0.003–1 μmol/kg) or the serotonin 5-HT_3_ receptor agonist phenylbiguanide (1–30 μg/kg; i.e., ~0.006–0.2 μmol/kg), according to Godlewski et al. (2003). CBD 10 mg/kg or its vehicle was injected *i.v.* 10 min before the first dose of capsaicin or phenylbiguanide.

#### Cardiovascular Effects of CBD in Pithed Rats

Two series (S_1_ and S_2_) of increasing doses of CBD (1, 3 and 10 mg/kg in each series) were injected *i.v.* to pithed rats. The particular doses of CBD were given with sufficient time for recovery to the pre-injection value (e.g., 10–15 min). The second series (S_2_) was examined in the presence of the nonselective β-adrenoceptor antagonist propranolol (1 μmol/kg; i.e., ~0.3 mg/kg) or the neuronal noradrenaline uptake inhibitor desipramine (0.1, 0.3, and 1 μmol/kg each; i.e., ~0.03, 0.09, and 0.3 mg/kg; examined in separate rats) or their vehicle given 5 min before CBD 1 mg/kg in S_2_. Two series (S_1_ and S_2_) of increasing doses of CBD were also examined in adrenalectomized and pithed WKY rats both in the presence and in the absence of propranolol 0.3 mg/kg.

### Preparation of Isolated Atria

Rats were anesthetized with pentobarbitone sodium (*i.p.*; 70 mg/kg). Hearts were removed, and left atria were dissected and suspended in an organ bath containing 10-ml Krebs solution of the following composition (mM): NaCl, 118; KCl, 4.8; MgSO_4_, 1; NaHCO_3_, 29, NaH_2_PO_4_ × 12H_2_O, 1; CaCl_2_, 2.25; glucose, 10; Na-pyruvate, 5; EDTA, 0.04 (pH, 7.4; 37°C). Each preparation was stretched to approximately 5 mN of force and allowed to equilibrate for 60 min. Atria were continuously stimulated by electrical field, applied using a bipolar platinum electrode with square waves (just over threshold, 5 ms duration, 2 Hz). Force of contractions was recorded using an isometric force transducer (PIM 100RE, BIO-SYS-TECH, Białystok, Poland). Tissue was allowed to equilibrate for 60 min. When steady basal values were obtained, concentration-response curves for CBD (1 nM–30 μM) were constructed.

### Drugs

Capsaicin, derivative of castor oil and ethylene oxide (Cremophor El), desipramine, dimethyl sulfoxide (DMSO), 1-phenylbiguanide (phenylbiguanide), propranolol, polyethylene glycol sorbitan monooleate (Tween 80), urethane (Sigma-Aldrich, Steinheim, Germany); (-)-cannabidiol (THC Pharm GmbH, Frankfurt, Germany and Tocris Cookson, Bristol, UK for *in vivo* and *in vitro* experiments, respectively); capsazepine (Tocris Cookson, Bristol, UK); pentobarbitone sodium (Biowet, Puławy, Poland). For experiments *in vivo*, drugs were dissolved in saline with the following exceptions: cannabidiol 3, 10, and 30 mg/kg was dissolved in a mixture of DMSO, Cremophor El, and saline (0.2:0.4:9.4; 0.3:1.4:8.3; and 0.45:2:7.55, respectively), urethane in water, capsaicin in a mixture of saline and ethanol (15:1), and capsazepine in a mixture of ethanol, Tween 80, DMSO, and saline (1:1:1:9.5). Saline and the vehicle for capsaicin did not affect the basal HR and BP. The vehicle for CBD 10 and 30 mg/kg and for capsazepine 0.4 mg/kg induced biphasic changes in cardiovascular parameters of anesthetized rats: a short-lasting (about 10 sec) bradycardia (by 15–20 beats/min) was followed by an increase in HR (by 15–30 beats/min) and SBP and DBP (by 15–20 mmHg in WKY and 25–30 mmHg in SHR) that lasted for about 5 min. The most evident change of all the above solvents in pithed rats was a short-lasting decrease in HR by about 20 beats/min. For experiments on *isolated atria*, cannabidiol was dissolved in DMSO. The final concentration of DMSO in the organ bath was 0.1% v/v; further dilutions were made with Krebs solution.

### Statistical Analysis

Results are given as means ± SEM of n experiments. Researchers were not blinded to the experimental conditions, but efforts were made to be close to the conditions of blinded assays, and our analysis did not include any subjective evaluation. Thus, (1) animals were treated in the same way; and (2) all data were obtained *via* direct recording of physiological parameters. Changes in cardiovascular parameters are shown as changes from baseline values. Positive inotropic effects of CBD in isolated atria are shown as a percentage of basal values. Statistical analysis was performed using Graph Pad Prism version 5.0 (La Jolla, CA, USA). For comparison of mean values, the t test for paired and unpaired data was used. To compare two compounds with the same control, the one-way analysis of variance (ANOVA) followed by the Dunnett post hoc test was used. The post hoc test was performed only if F was significant. Telemetry data were analyzed using repeated two-way analysis of variance (ANOVA) with the Bonferroni post hoc test. A value of *p* < 0.05 was considered statistically significant. To determine the maximal effects (E_max_) and the potency of CBD in isolated atria, the pEC_50_ values (the negative logarithm of the concentration causing the half-maximum effect) were determined from the individual concentration-response curves.

## Results

### Influence of CBD on Pithed and Vagotomized Rats

In all rats which were later allocated to the *in situ* (pithed rats), *in vivo* (anesthetized rats), and *in vitro* groups (isolated rat atria), basal SBP and HR were recorded by the noninvasive tail-cuff method. SBP was higher in SHR (178 ± 3 mmHg; *n* = 64) than in WKY (110 ± 2 mmHg; *n* = 107) (*p* < 0.001); the same held true for HR, which was 365 ± 5 vs 331 ± 2 beats/min (*p* < 0.001), respectively.

As shown in Table 1, SBP and DBP were diminished in pithed and vagotomized rats in comparison to anesthetized animals. Propranolol 0.3 mg/kg decreased basal HR by about 10% in SHR and tended to do so in WKY (compare S_1_ and S_2_ in Table 1). SBP and DBP were increased by desipramine 0.09 mg/kg by about 20% and by desipramine 0.3 mg/kg by about 35%; in addition, the highest dose of desipramine enhanced HR by 5% (Table 1). Bilateral adrenalectomy led to a further decrease in all cardiovascular parameters in pithed rats (Table 1).

**Table 1:** Basal systolic (SBP) and diastolic (DBP) blood pressure and heart rate (HR) immediately before administration of cannabidiol in urethane-anesthetized and in pithed normotensive Wistar-Kyoto (WKY) and spontaneously hypertensive (SHR) rats.

Group		n		WKY			n	SHR		
				SBP (mmHg)	DBP (mmHg)	HR (beats/min)		SBP (mmHg)	DBP (mmHg)	HR (beats/min)
Anesthetized rats (before CBD 10 mg/kg)	All controls	22		104 ± 2	52 ± 2	284 ± 6	20	131 ± 3^***^	66 ± 3^***^	332 ± 6^***^
	Controls with comparable basal values^1^	8		114 ± 3	61 ± 3	308 ± 11	10	122 ± 3	62 ± 3	319 ± 6
	Vagotomy	5		107 ± 3	47 ± 3	390 ± 10	5	124 ± 3	52 ± 3	428 ± 11
	After CBD 30 mg/kg	5		103 ± 3	41 ± 5	255 ± 3	5	139 ± 5^***^	68 ± 5^**^	341 ± 7^***^
	Vehicle for capsazepine	5		113 ± 6	63 ± 5	295 ± 19	5	147 ± 4^**^	78 ± 5	322 ± 14
	Capsazepine 0.4 mg/kg	5		110 ± 6	63 ± 7	294 ± 7	5	131 ± 5^*^	77 ± 6	361 ± 20^*^
Pithed rats^2^ (before CBD 1 mg/kg)	Control (vehicle for propranolol)	6	S_1_ S_2_	55 ± 4 54 ± 4	33 ± 3 32 ± 3	279 ± 5 266 ± 9^##^	5	60 ± 5 56 ± 5	35 ± 4 33 ± 3	304 ± 11 285 ± 14
	Propranolol 0.3 mg/kg	5	S_1_ S_2_	54 ± 6 54 ± 6	32 ± 4 32 ± 4	281 ± 5 253 ± 12	5	59 ± 4 59 ± 4	35 ± 2 35 ± 3	311 ± 7 284 ± 5^##^
	Vehicle for desipramine	5	S_1_ S_2_	51 ± 2 52 ± 2	27 ± 1 27 ± 2	300 ± 10 277 ± 12^##^				
	Desipramine 0.03 mg/kg	6	S_1_ S_2_	53 ± 2 57 ± 3	30 ± 1 31 ± 2	307 ± 10 278 ± 13				
	Desipramine 0.09 mg/kg	6	S_1_ S_2_	57 ± 3 69 ± 3^###^	32 ± 2 38 ± 3^##^	305 ± 6 308 ± 6				
	Desipramine 0.3 mg/kg	6	S_1_ S_2_	53 ± 3 71 ± 6^##^	30 ± 2 42 ± 5^#^	285 ± 9 298 ± 10^#^				
Pithed and adrenalectomized rats^3^ (before CBD 1 mg/kg)	Control (vehicle for propranolol)	6	S_1_ S_2_	36 ± 2 35 ± 2	26 ± 1 26 ± 1	257 ± 7 227 ± 10^##^				
	Propranolol 0.3 mg/kg	5	S_1_ S_2_	42 ± 2 37 ± 2^##^	23 ± 1 19 ± 1^##^	264 ± 11 253 ± 13^#^				

^*^p < 0.05; ^**^p< 0.01; ^***^p < 0.001 compared to the respective values in WKY (t test for unpaired data); ^#^p < 0.05; ^##^p < 0.01; ^###^p < 0.001 compared to the respective values in S_1_ (t test for paired data).

1 To have similar basal levels of blood pressure and heart rate in normo- and hypertensive rats, the 8 WKY with the highest and the 10 SHR with the lowest levels of SBP, DBP and HR were considered.

2 Cannabidiol was given twice (S_1_, S_2_); propranolol, desipramine (or their solvents) were administered between S_1_ and S_2_.

3 Adrenalectomy was performed before S_1_.

**Figure 1:**
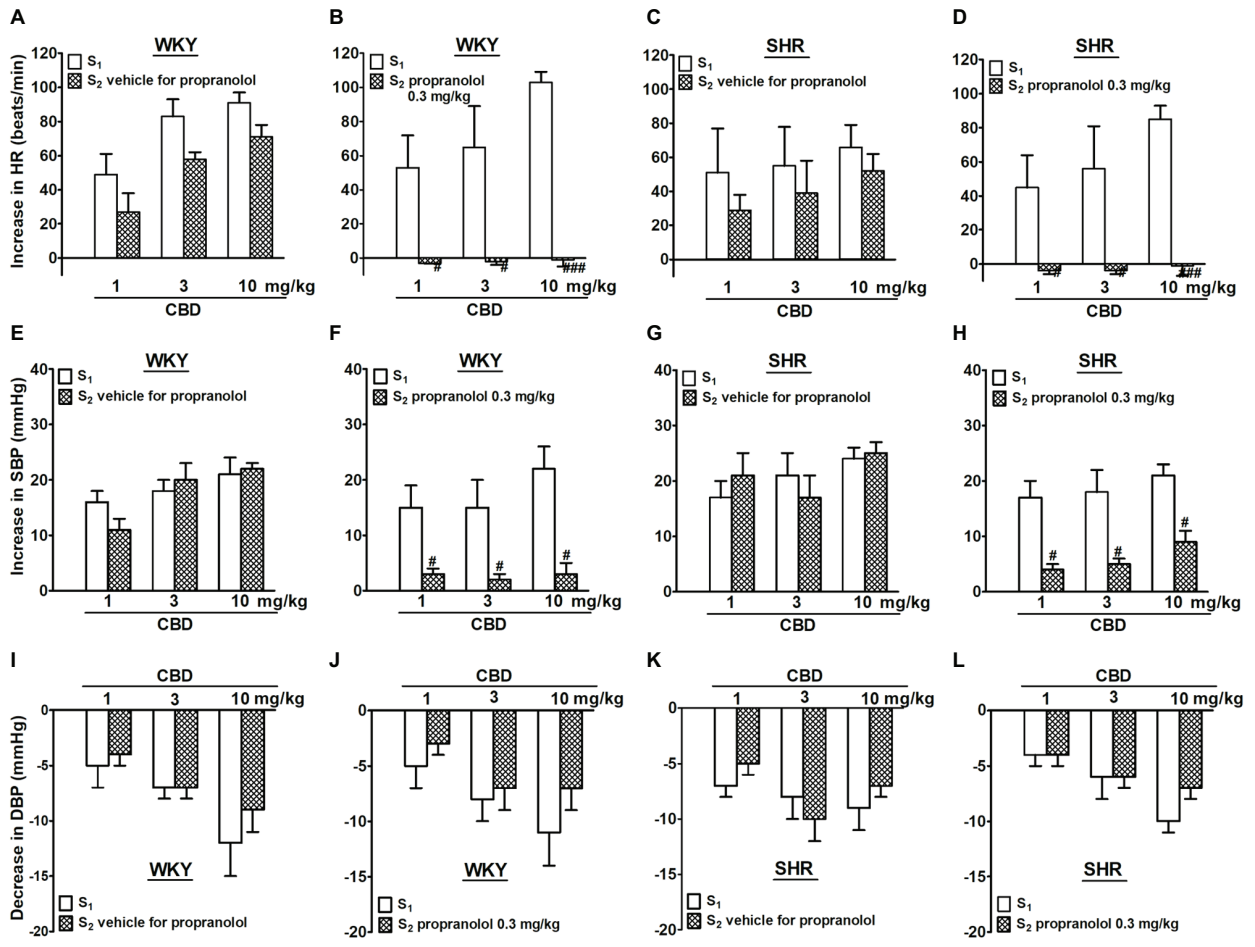
Influence of propranolol 0.3 mg/kg **(B,D,F,H,J,L)** and its vehicle **(A,C,E,G,I,K)** on changes in heart rate (HR; **A–D**) and systolic (SBP; **E–H**) and diastolic (DBP; **I–L**) blood pressure elicited by cannabidiol (CBD) in urethane-anesthetized and pithed normotensive Wistar-Kyoto (WKY) and spontaneously hypertensive (SHR) rats. Data are given as the means ± SEM of 5–6 rats. For comparison of mean values, the t test for paired data was used. ^#^*p* < 0.05; ^###^*p* < 0.001 in comparison to the respective values before administration of propranolol (S_1_).

**Figure 2:**
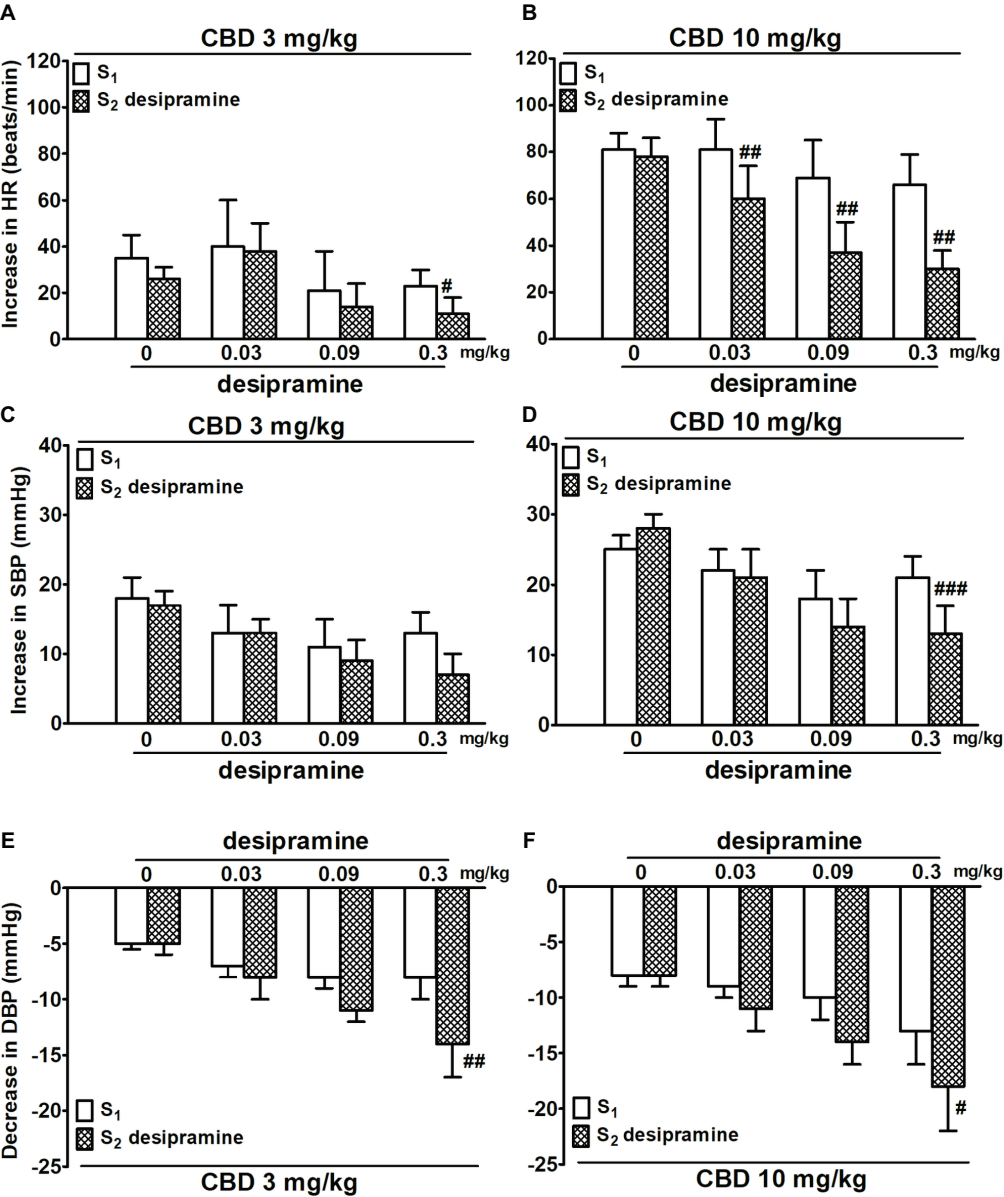
Influence of desipramine (0.03, 0.09 and 0.3 mg/kg) or its vehicle (0) on the cannabidiol [CBD 3 **(A,C,E)** and 10 **(B,D,F)** mg/kg]-induced changes in heart rate (HR; **A,B**) and systolic (SBP; **C,D**) and diastolic (DBP; **E,F**) blood pressure in urethane-anesthetized and pithed normotensive Wistar-Kyoto rats (WKY). Data are given as the means ± SEM of 5–6 rats. For comparison of mean values, the t test for paired data was used. ^#^*p* < 0.05; ^##^*p* < 0.01; ^###^*p* < 0.001 in comparison to the respective values before administration of desipramine (S_1_).

CBD 1, 3, and 10 mg/kg given *i.v.* to the same rat increased HR and SBP by 50–100 beats/min and about 20 mmHg, respectively; comparable effects were obtained in WKY and SHR (Figures 1, 2). Compared to the first administration of CBD (S_1_), the second one (S_2_) induced slightly lower increases in HR (by about 20 beats/min) without affecting pressor responses. In contrast to SBP, DBP was dose-dependently decreased by 5–10 mmHg following administration of CBD 1, 3, and 10 mg/kg. The effect was comparable in normotensive vs hypertensive rats and upon the first vs the second CBD injection (Figures 1I–L, 2E,F). Propranolol 0.3 mg/kg given 5 min before S_2_ completely prevented the CBD-induced tachycardia and strongly reduced the increase in SBP without affecting the decrease in DBP both in WKY and SHR (Figure 1).

Desipramine (0.03, 0.09, and 0.3 mg/kg and studied in WKY only) reduced the increase in HR elicited by CBD 10 mg/kg by 20–30 beats/min (Figure 2B). In addition, the highest dose of desipramine reduced the increase in HR induced by CBD 3 mg/kg (Figure 2A) and the increase in SBP induced by CBD 10 mg/kg (Figure 2D) but enhanced the fall in DBP elicited by CBD 3 and 10 mg/kg (Figures 2E,F).

**Figure 3:**
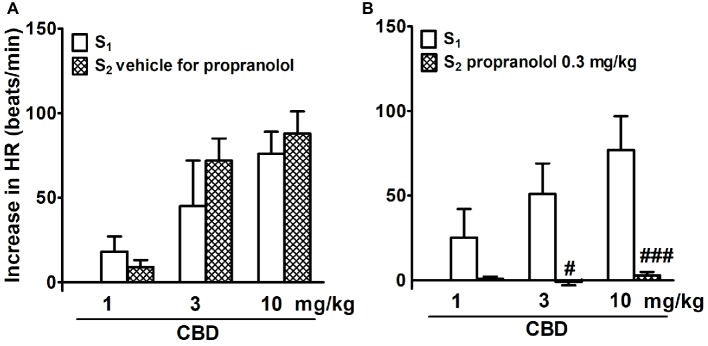
Influence of propranolol 0.3 mg/kg **(B)** and its vehicle **(A)** on changes in heart rate (HR) elicited by cannabidiol (CBD) in urethane-anesthetized, pithed and adrenalectomized normotensive Wistar-Kyoto (WKY) and spontaneously hypertensive (SHR) rats. Data are given as the means ± SEM of 5–6 rats. For comparison of mean values, the t test for paired data was used. ^#^*p* < 0.05; ^###^*p* < 0.001 in comparison to the respective values before administration of propranolol (S_1_).

In adrenalectomized, pithed, and vagotomized WKY rats, CBD 1, 3, and 10 mg/kg dose dependently increased HR by 20–70 beats/min; effects were similar during S_1_ and S_2_ (Figure 3A). Propranolol completely prevented the CBD-induced tachycardia (Figure 3B). Because of the low basal BP, changes in BP in response to CBD were too small to be calculated.

### Influence of CBD on Anesthetized Rats

SBP and HR were lower in anesthetized than in conscious SHR (by 25 and 10%, respectively; *p* < 0.001; compare values in Table 1 and in the “Influence of CBD on Pithed and Vagotomized Rats” section). In WKY, anesthesia only decreased HR (by about 15%; *p* < 0.001) without affecting SBP. Vagotomy increased HR (but not BP) in anesthetized WKY and SHR by about 35 and 30%, respectively (*p* < 0.001). Capsazepine did not affect BP and HR in anesthetized animals.

**Figure 4:**
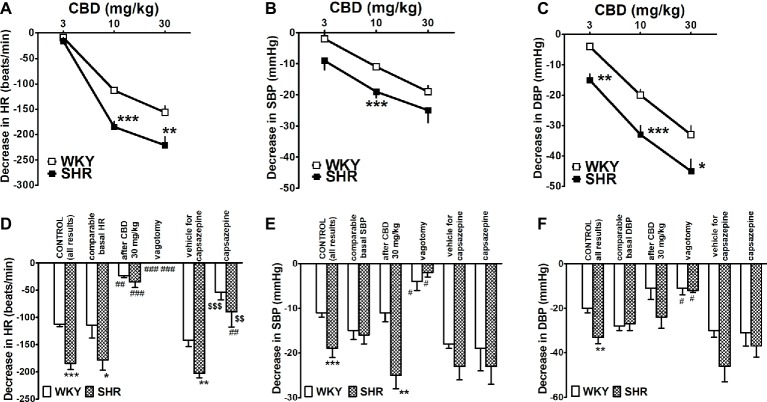
Influence of intravenous injection of cannabidiol (CBD) on heart rate (HR; **A,D**) and systolic (SBP; **B,E**) and diastolic (DBP; **C,F**) blood pressure in urethane-anesthetized normotensive Wistar-Kyoto (WKY) and spontaneously hypertensive (SHR) rats. With one exception all doses of CBD were examined in separate animals. Changes were determined under the following conditions, i.e., in all rats (CONTROL); in rats with comparable basal values (for explanation, see text or legend to Table 1); when CBD 10 mg/kg was given after CBD 30 mg/kg; after vagotomy; and in the presence of capsazepine 0.4 mg/kg or its vehicle. Data are given as the means ± SEM of 5–22 rats. We used the t test for unpaired data (* and $) and the one-way analysis of variance (ANOVA) followed by the Dunnett post hoc test (#). *p < 0.05; **p < 0.01; ***p < 0.001 in comparison to the respective values in WKY; ^#^*p* < 0.05; ^##^*p* < 0.01; ^###^*p* < 0.001 in comparison to the respective CONTROL group; ^$$^*p* < 0.01; ^$$$^*p* < 0.001 in comparison to the respective group treated with vehicle for capsazepine.

Rapid injection of CBD (3, 10 and 30 mg/kg) caused dose-dependent decreases in HR, SBP and DBP in anesthetized rats (Figures 4A–C). The highest dose of CBD diminished HR and DBP in SHR by about 65% of basal values and SBP by about 20%. Importantly, all cardiovascular responses to CBD 10 and 30 mg/kg were smaller by 30–40% in WKY than in SHR. The maximal changes in HR and BP were obtained both for SHR and WKY within the first 10 and 20–30 s, respectively. They lasted for about 20 (HR in both strains), 65 (SBP and DBP in SHR), and 100–135 s (SBP and DBP in WKY). In order to ensure comparable experimental conditions and to exclude the possibility that the lower response to CBD in WKY is related to lower basal values in these animals (see above), we compared responses to CBD 10 mg/kg in WKY and SHR with particular high and low basal HR and BP, respectively. However, in spite of a comparable level of HR and BP (for respective basal values, see Table 1), the bradycardia induced by CBD 10 mg/kg was still higher in SHR than in WKY (Figure 4D), whereas the decrease in SBP or DBP did not differ any longer (Figures 4E,F).

During the course of our study, we observed that rapid desensitization against CBD occurred in the case of HR. For example, the bradycardia elicited by CBD 10 mg/kg was almost completely reduced when CBD 10 mg/kg was given after CBD 30 mg/kg both in SHR and in WKY (Figure 4D). For this reason, each dose of CBD was injected to separate rats in subsequent experiments. Bilateral vagotomy prevented or strongly diminished the cardiovascular responses to CBD 10 mg/kg in both strains (Figures 4D–F), whereas capsazepine 0.4 mg/kg only strongly reduced its effect on HR (Figure 4D) without affecting its effect on BP (Figures 4E,F).

Interestingly, the CBD-induced bradycardia consisted of two phases. The second one that lasted for about 5 min occurred immediately after the first one and was slower. Thus, the maximal decreases were reached within 2 min. They amounted to 36 ± 5 (*n* = 22) and 27 ± 4 (*n* = 20) beats/min in WKY and SHR, respectively. They were not modified by vagotomy and by capsazepine (data not shown).

**Figure 5:**
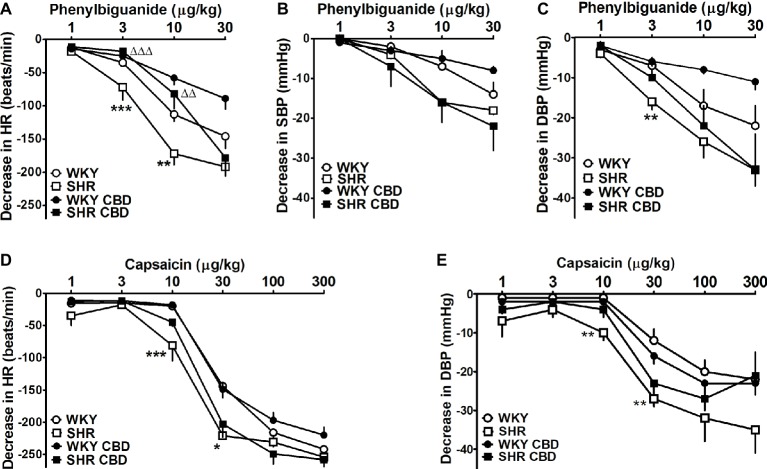
Influence of intravenous injection of cannabidiol (CBD) 10 mg/kg or its vehicle on decreases in heart rate (HR; **A,D**) and systolic (SBP; **B**) and diastolic (DBP; **C,E**) blood pressure induced by bolus injection of phenylbiguanide **(A–C)** and capsaicin **(D,E)** in urethane-anesthetized normotensive Wistar-Kyoto (WKY) and spontaneously hypertensive (SHR) rats. Data are given as the means ± SEM of 6–11 rats. We used the t test for unpaired data. *p < 0.01; **p < 0.01; ***p < 0.001 in comparison to the respective values in WKY; ^ΔΔ^*p* < 0.01; ^ΔΔΔ^*p* < 0.001 in comparison to the respective group treated with vehicle for CBD.

The short-lived fall in HR elicited by CBD reminds of the Bezold-Jarisch reflex. In subsequent experiments, another two compounds eliciting this reflex were studied. Bolus injection of the 5-HT_3_ and TRPV1 receptor agonists, phenylbiguanide (1–30 μg/kg), and capsaicin (1–300 μg/kg) induced short-lasting dose-dependent rapid decreases in HR, SBP, and DBP in normotensive and hypertensive rats (Figure 5). The effect on HR was the most marked response in either strain and amounted to about 200 and 260 beats/min after injection of phenylbiguanide and capsaicin, respectively. By contrast, decreases in SBP after capsaicin injection were too small to be calculated and are not shown in Figure 5. Both the phenylbiguanide- and capsaicin-elicited bradycardia and hypotension were more marked by 40–60 beats/min and 10–15 mmHg (for 3 and 10 μg/kg and 10 and 30 μg/kg, respectively) in SHR than in WKY (Figure 5). In SHR, CBD 10 mg/kg diminished the phenylbiguanide-induced bradycardia to the level observed in WKY (Figure 5A) and tended to diminish the decrease in DBP (Figure 5C). By contrast, the same dose of CBD failed to modify the reflex bradycardia and hypotension elicited by capsaicin (Figures 5D,E).

### Influence of CBD on Conscious Rats

**Figure 6:**
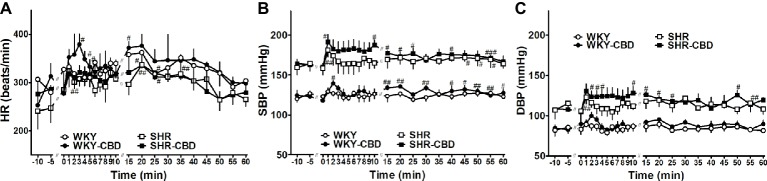
Telemetered heart rate (HR, **A)**, systolic (SBP, **B)** and diastolic (DBP, **C)** blood pressure in normotensive Wistar-Kyoto (WKY) and spontaneously hypertensive (SHR) rats before and after intraperitoneal injection with cannabidiol (CBD) 10 mg/kg over a time period of about 30 sec or its vehicle at time 0. Data are given as the means ± SEM of 4 rats per group. They were analysed by repeated two-way analysis of variance (ANOVA) with the Bonferroni post hoc test. All values of SBP and DBP in SHR (in the presence of CBD or its vehicle) were higher than their respective values in WKY (*p* < 0.01). ^#^*p* < 0.05; ^##^*p* < 0.01; ^###^*p* < 0.001 vs the respective value at time 0.

Seven days after transmitter implantation basal SBP in SHR (159 ± 3 mmHg; *n* = 8) was higher than in WKY (121 ± 4 mmHg; n = 8) (*p* < 0.001). The same held true for DBP, which was 105 ± 4 mmHg in SHR and 86 ± 5 mmHg in WKY (*p* < 0.05), whereas HR (282 ± 8 vs 303 ± 21 beats/min, respectively) did not differ between both groups. As shown in Figure 6, administration of CBD 10 mg/kg or its vehicle increased all cardiovascular parameters during the first 5 min immediately after their application. Although CBD tended to increase HR (by about 60 beats/min in WKY) and SBP and DBP (by 15–20 mmHg in SHR and by 10–15 mmHg in WKY) more markedly than its vehicle, none of the differences reached a significant level.

### Influence of CBD on Isolated Rat Atria

**Figure 7:**
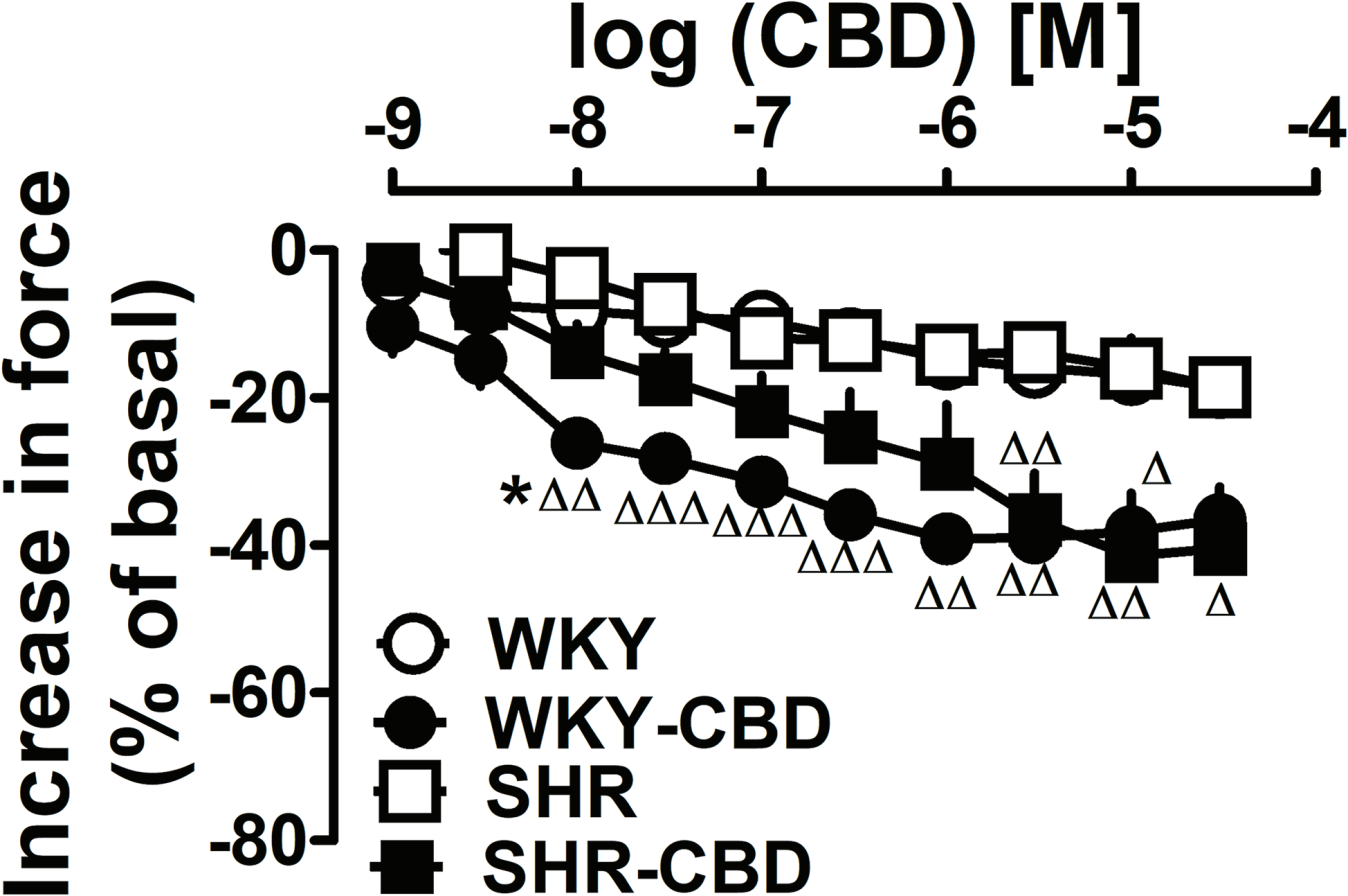
Influence of cannabidiol (CBD) or its vehicle on contractile force of left atria isolated from normotensive Wistar-Kyoto (WKY) and spontaneously hypertensive (SHR) rats. Data are given as the means ± SEM of 5–6 rats. They were analysed by the one-way analysis of variance (ANOVA) followed by the Dunnett post hoc test. ^Δ^*p* < 0.05; ^ΔΔ^*p* < 0.01; ^ΔΔΔ^*p* < 0.001 in comparison to the respective values without CBD. ^*^p < 0.05 in comparison to the respective value in SHR.

We did not observe any differences between basal values of atria isolated from WKY and later treated with CBD or its vehicle and from SHR and later treated with CBD or its vehicle (*n* = 5 each). Thus, basal force was 2.3 ± 0.1; 2.2 ± 0.1; 2.3 ± 0.1; and 2.3 ± 0.1 mN, respectively. As shown in Figure 7, the final concentration of DMSO (vehicle for CBD) reduced contractility by 20% (Figure 7). CBD 1 nM–30 μM decreased atrial contractility by up to another 20% both in WKY and SHR (Figure 7). E_max_ for atria isolated from WKY treated with CBD or its vehicle and from SHR treated with CBD or its vehicle were −19 ± 1 and −39 ± 2 (*p* < 0.001) and −19 ± 3 and −41 ± 9 (*p* < 0.05). The pEC_50_ value for CBD in WKY (8.0 ± 0.2) was higher (*p* < 0.05) than that in SHR (6.8 ± 0.3).

## Discussion

### General

The aim of our study was to identify cardiovascular effects of cannabidiol in conscious, urethane-anesthetized, and pithed rats and to compare them in normotensive WKY and in SHR (the most frequently studied genetic model of primary hypertension; Pinto et al., 1998). Indeed, both conscious and anesthetized SHR had higher BP than their WKY counterparts. We preferred urethane over pentobarbitone anesthesia since urethane, unlike pentobarbitone, anesthesia does not affect reflex responses (Maggi and Meli, 1986) and moreover since CBD 10 mg/kg interferes with pentobarbitone anesthesia in mice (Chesher et al., 1974). Pithed rats, i.e., animals in which the central nervous system is destroyed by a steel rod, allowed us to study peripheral effects only. In the experiments in which the influence of CBD on the TRPV1 and 5-HT_3_ receptor-mediated Bezold-Jarisch reflex (Campagna and Carter, 2003) was examined, the latter reflex was elicited by the respective agonists capsaicin and phenylbiguanide. CBD was mainly used at 10 mg/kg, since this dose acutely diminished the stress-induced increases in BP and HR in rats (Resstel et al., 2009) and in humans (i.e., ~600 mg/70 kg; Jadoon et al., 2017) and chronic administration of this dose to rats attenuated the left ventricular cardiac dysfunction induced by diabetes (Rajesh et al., 2010) and doxorubicin (Hao et al., 2015) and improved the endothelium-dependent vasorelaxation in mesenteric arteries of diabetic animals (Wheal et al., 2017).

### Cardiovascular Effects of CBD in Pithed Rats and *in vitro*

In pithed rats, CBD (1, 3, and 10 mg/kg; *i.v.*) dose-dependently increased HR and SBP, maximally by about 90 beats/min and 20 mmHg, respectively. The second administration of the same dose of CBD to the same rat led to comparable increases in both cardiovascular parameters. The CBD-induced changes were similar in SHR and WKY. Thus, we only performed the initial two series of experiments on both strains. The CBD-induced increases in HR and SBP were fully or very markedly suppressed by the nonselective β-adrenoceptor antagonist propranolol 0.3 mg/kg, suggesting a direct or indirect sympathomimetic activity of CBD.

Its effect might be related to the activation of postsynaptic ß-adrenoceptors but this possibility can be excluded since CBD has no sufficient ß-adrenoceptor affinity as shown in binding (Hillard and Bloom, 1982) and functional experiments (Weresa et al., 2018). The possibility that CBD leads to an increased *exocytotic* noradrenaline release *via* a presynaptic site like in the study by Pertwee et al. (2002) can also be discarded since propagated neuronal activity does not occur in the pithed rat model (unless electrical stimulation is carried out). A third possibility, namely a stimulatory effect of CBD on catecholamine release in the adrenal medulla like that of histamine via H_1_ receptors (Hill et al., 1997), can also be excluded since the effect of CBD on HR was not substantially affected in adrenalectomized rats.

On the other hand, the noradrenaline reuptake inhibitor desipramine (0.03, 0.09 and 0.3 mg/kg) dose-dependently diminished the enhancement in HR and SBP elicited by CBD 3 and/or 10 mg/kg, suggesting that CBD causes *carrier-mediated* noradrenaline release from the sympathetic nerve endings or inhibits reuptake of noradrenaline, thereby leading to an increased effect of this monoamine. High CBD concentrations (10 and 100 μM) have indeed elicited spontaneous noradrenaline release in rat hypothalamic synaptosomes (Poddar and Dewey, 1980), whereas in our previous study in the rat and mouse renal cortex (Ilayan et al., 2013), CBD 10 μM did not show such a tyramine-like effect. CBD also possesses an inhibitory effect on noradrenaline reuptake (Poddar and Dewey, 1980), and this property may represent the most plausible mechanism to account for the effect of CBD on HR and SBP.

Two findings deserve separate discussion. First, the stimulatory effect of CBD on SBP was relatively small and, in adrenalectomized animals, it was so small that it could not even be quantified. The reason may be that CBD has a combined stimulatory and inhibitory effect on myocardial contractile force. Indeed, our *in vitro* experiments show that CBD decreased the force of stimulated left atria, thereby confirming the study by Ali et al. (2015) in which a negative inotropic effect of CBD 1 μM was described on rat ventricular myocytes. In our hands, the potency of CBD for this effect was more marked in WKY than in SHR, whereas the maximum effect of about 20% did not show a difference.

Second, the question arises why CBD increased SBP but decreased DBP. In this context, one should keep in mind that SBP and DBP are dependent mainly on heart function and peripheral resistance, respectively. Thus, the strong positive chronotropic effect of CBD is probably responsive for the increase in SBP whereas the decrease in DBP results from a direct vasodilatory effect of CBD previously described under *in vitro* conditions (Stanley et al., 2013) and from its negative inotropic effect (see above). Indeed, propranolol strongly inhibited the CBD-induced increase in SBP, but did not affect the decrease in DBP. With respect to desipramine 0.3 mg/kg, the CBD 10 mg/kg-related increase in SBP was reduced but the decrease in DBP was even enhanced. The reason for the latter effect may be that desipramine enhanced basal BP, and that under this condition, the vasodilatory effect of CBD was more marked.

### Cardiovascular Effects of CBD in Anesthetized Rats

Rapid *i.v.* injection of CBD (3, 10 and 30 mg/kg) to urethane-anesthetized rats dose-dependently decreased HR, SBP, and DBP. The CBD-induced bradycardia is a reflex response, which was completely abolished by bilateral vagotomy. The effect of CBD resembles the cardiovascular responses typical for the capsaicin-induced and phenylbiguanide-induced (present paper) and anandamide-induced and methanandamide-induced (Malinowska et al., 2001) Bezold-Jarisch reflex. Indeed, responses to all agonists were immediate and short-lasting. Moreover, comparable decreases in HR and DBP (by about 250 beats/min and 40 mmHg) have been obtained for the highest doses of agonists, i.e., 30 mg/kg, 300 and 30 μg/kg, and 1 and 10 mg/kg, respectively. It seems that the CBD-induced bradycardia is mediated *via* TRPV1 receptors, since it was strongly inhibited (by about 60%) by the TRPV1 receptor antagonist capsazepine 0.4 mg/kg. One may argue that only one dose of capsazepine was used and that this compound has limited selectivity as a TRPV1 antagonist (Tabrizi et al., 2017). First, in urethane-anesthetized rats, capsazepine 0.13, 0.4, and 1.3 mg/kg inhibited the TRPV1-mediated reflex bradycardia induced by capsaicin, anandamide, or methanandamide by up to 50% and the maximum occurred at 0.4 mg/kg (Malinowska et al., 2001), i.e., the dose used in the present study. Second, in segments of adult rat vagus nerves, capsazepine competitively inhibited the capsaicin-induced efflux of [14C]-guanidinium without affecting that induced by depolarization (50 mM KCl) (Bevan et al., 1992). Third, in urethane-anaesthetized rats, capsazepine 0.3 mg/kg/min completely inhibited the cardiorespiratory reflexes induced by capsaicin but only partially reduced those elicited by 2-aminoethoxydiphenyl borate (a common activator of TRPV 1, 2, and 3 channels) (Gu et al., 2005).

The effects of CBD, capsaicin, and phenylbiguanide were higher in SHR than in WKY. Analysis of the effects of CBD in SHR and WKY with comparable basal cardiovascular responses allowed us to exclude the possibility that higher responses to CBD in SHR resulted from higher basal values of cardiovascular parameters in SHR. By contrast, the serotonin- (Thomas et al., 1998) and the phenylbiguanide- (Uggere et al., 2000) induced reflex bradycardia were lower in conscious SHR than in conscious WKY. In the study by McQueen et al. (2004) in pentobarbitone-anesthetised rats, CBD did not elicit the vasorespiratory effects previously described for the TRPV1 receptor agonists capsaicin and anandamide. However, CBD was used at doses up to 2 mg/kg that were also ineffective in our study.

We found that the CBD-induced bradycardia underwent tachyphylaxis, i.e., only one dose of CBD could be administered to one rat. However, CBD 10 mg/kg failed to modify (desensitize) the capsaicin-induced Bezold-Jarisch reflex. On the other hand, it has been shown that CBD desensitizes TRPV1 receptors to capsaicin in HEK293 cells (Bisogno et al., 2001; De Petrocellis et al., 2011; Iannotti et al., 2014). It is not clear whether the acute desensitization and tachyphylaxis of TRPV1 receptors share the same cellular mechanisms (Vyklický et al., 2008). Moreover, De Petrocellis et al. (2011) argue that one of the limitations of their study is the fact that they did not perform their experiments in a model with native TRPV1 receptors, such as, for example, sensory neurones. We did not find any desensitization/tachyphylaxis in the case of the TRPV1-mediated reflex bradycardia induced by capsaicin or anandamide (present study; Malinowska et al., 2001) probably pointing to different channel regions of the TRPV1 receptor addressed by CBD on the one hand and by capsaicin on the other. Similarly, the activation and desensitization of recombinant TRPV1 receptors in HEK293 cells by camphor appear to occur *via* a mechanism distinct from that involved in the case of capsaicin (Xu et al., 2005).

The question arises why the decrease in BP induced by CBD 10 mg/kg was only partially attenuated by vagotomy and not affected at all by capsazepine or repeated CBD. Firstly, one should keep in mind that the fall in BP results from the Bezold-Jarisch reflex-elicited decrease in HR but not from a direct influence of the agonists on blood vessels (Malinowska et al., 2001) and that capsazepine has only moderate potency at TRPV1 receptors (Szallasi and Blumberg, 1999; note that in the study by Malinowska et al. (2001) the TRPV1 receptor-agonist-related decrease in BP was also not consistently antagonized by capsazepine). Secondly, as mentioned above, CBD is known to possess vasodilatory (Stanley et al., 2013) and negative inotropic (present study) activities that may be responsible for a vasodepressor effect of this compound independent of the Bezold-Jarisch reflex.

We have also found that CBD 10 mg/kg inhibited the reflex bradycardia induced by the 5-HT_3_ receptor agonist phenylbiguanide in SHR and tended to do so in WKY. An inhibitory effect of CBD 1 μM has so far only been shown for recombinant 5-HT_3_ receptors in HEK293 cells (Xiong et al., 2011) and *Xenopus laevis* oocytes (Yang et al., 2010). 5-HT_3_ receptor antagonists are well-established powerful antiemetic drugs; preclinical studies suggest that CBD possesses effectiveness against vomiting as well (Sharkey et al., 2014).

Another question is why the CBD-induced tachycardia and pressor effect obtained in pithed and vagotomized rats did not occur in anesthetized animals. As mentioned above, vagotomy excludes reflex responses to CBD, and pithing allows to examine its peripheral effects only. One may assume that CBD counteracts its peripheral sympathomimetic properties *via* a central influence. However, CBD microinjected into the stria terminalis (Alves et al., 2010; Gomes et al., 2013) or into the cisterna magna (Granjeiro et al., 2011) did not affect basal HR and BP of unrestrained rats. Potential effects of CBD on other areas responsible for the central regulation of cardiovascular system such as rostral ventrolateral medulla, paraventricular nucleus of hypothalamus, or nucleus tractus solitarii have so far not been examined.

### Cardiovascular Effects of CBD in Conscious Rats

In the experiments on unrestrained conscious rats in which cardiovascular parameters were determined telemetrically and CBD 10 mg/kg was slowly infused *i.p*., an antihypertensive activity of the cannabinoid as expected by several authors (Booz, 2011; Stanley et al., 2013; Sultan et al., 2017) did not occur. The only effect we obtained was an increase in all cardiovascular parameters immediately after administration of CBD or its vehicle, i.e., the typical stress reaction for injection. CBD led to more marked increases in HR and SBP than vehicle although the difference did not reach a significant level. CBD is known to reduce BP and HR under stressful conditions (see Introduction) but its microinjection into the BNST increased HR to acute restraint stress (Gomes et al., 2013). The failure to observe the CBD-induced Bezold-Jarisch reflex in conscious rats is not surprising since this reflex can be induced by rapid injection only.

## Conclusions

**Figure 8:**
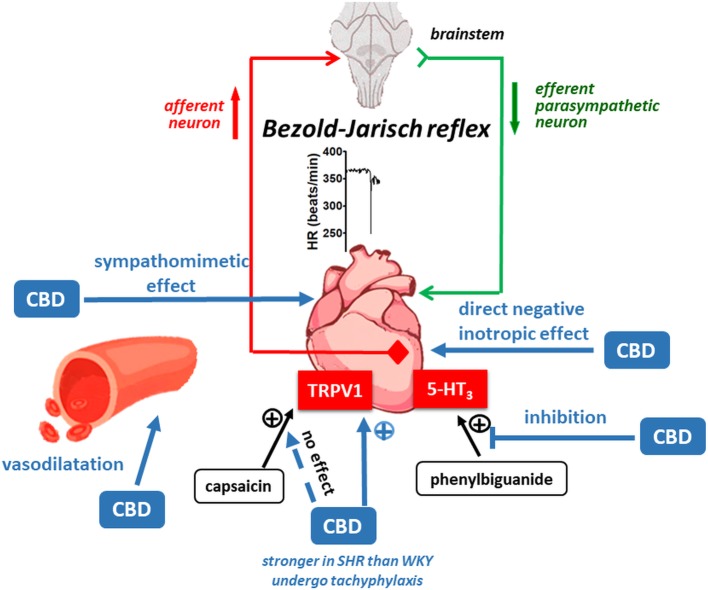
Possible mechanisms involved in the cardiovascular effects of cannabidiol (CBD) in normotensive Wistar-Kyoto (WKY) and spontaneously hypertensive (SHR) rats.

Our study shows (Figure 8) that cannabidiol (1) induces reflex bradycardia (the Bezold-Jarisch reflex) likely via TRPV1 receptors (which undergo tachyphylaxis) more markedly in SHR than in WKY; (2) inhibits the 5-HT_3_ but not the TRPV1 receptor-related Bezold-Jarisch reflex; (3) has peripheral sympathomimetic, (4) vasodilatory, and (5) negative inotropic effects. Although an antihypertensive effect of CBD suggested by other investigators could not be shown in conscious rats with telemetric determination of blood pressure, the above properties of CBD should be taken under consideration when CBD is used for therapeutic purposes (see Introduction).

## Data Availability

All datasets generated for this study are included in the manuscript and/or the supplementary files.

## Ethics Statement

All surgical procedures and experimental protocols were performed in accordance with the European Directive (2010/63/EU) and Polish legislation. The study was approved by the local Animal Ethics Committee in Olsztyn (Poland, nr 40/2016, 80/2017).

## Author Contributions

RK, MT, and JW performed the experiments, analyzed and interpreted the data. BM and ES designed the study and wrote the manuscript. All authors gave final approval of the current version of the article being submitted.

### Conflict of Interest Statement

The authors declare that the research was conducted in the absence of any commercial or financial relationships that could be construed as a potential conflict of interest.

## Abbreviations

5-HT_3_: serotonin 5-HT_3_ receptors
BNST: bed nucleus of the stria terminalis
CBD: cannabidiol
DBP: diastolic blood pressure
HR: heart rate
SBP: systolic blood pressure
TRPV1: transient receptor potential vanilloid type 1.
